# The impact of information technology applications on the quality of life of disabled older adults in nursing homes in China: a qualitative study

**DOI:** 10.3389/fpubh.2025.1560306

**Published:** 2025-04-07

**Authors:** Hong Zhang, Xiaoyan Liao, Shuang Liang, Lifang Tong, Jun Shen, Bin Peng, Lin Wu, Lu Gao, Qianying Jia, Liu Ren, Lanyue Luo, Yixin Wang, Xiaoli Zhang

**Affiliations:** ^1^Department of Nursing, The First Affiliated Hospital of Chongqing Medical University, Chongqing, China; ^2^Chongqing Nursing Vocational College, Chongqing, China; ^3^School of Public Health, Chongqing Medical University, Chongqing, China; ^4^Chongqing Jianzhu College, Chongqing, China; ^5^Chongqing University of Chinese Medicine, Chongqing, China; ^6^The People’s Hospital of Tongliang District, Chongqing, China

**Keywords:** information technology, nursing homes, disabled older adults, quality of life, qualitative study

## Abstract

**Objective:**

China’s smart aging policy system has been evolving to become more comprehensive, continuously promoting the application of information technology in nursing homes. This study explores the adaptation process and experiences of disabled older adults with the use of information technology in nursing homes from four perspectives: physiological, psychological, social, and environmental, and examines its impact on their quality of life.

**Methods:**

An interpretative phenomenological approach was adopted, with purposive sampling used to recruit participants. Semi-structured interviews were conducted with 14 disabled older adults, and the data were analyzed using Van Manen’s phenomenology of practice method.

**Results:**

Four main themes and 16 sub-themes were identified: *Physical Health and Functional Capacity:* subjective health perception, physical functioning, chronic disease management, sleep quality, and nutritional status; *Psychological Wellbeing and Emotional Support:* attitudes toward aging, negative emotions, emotional companionship, and sense of meaning in life; *Social Relationships and Social Engagement:* interactions with family and friends, participation in social activities, social roles, and social support; and *Environmental Adaptation and Digital Challenges:* safety and comfort of the living environment, ease of independent mobility, the ‘digital divide’, and protection of personal privacy and data.

**Conclusion:**

The application of information technology in nursing homes in China has partially resolved longstanding issues in traditional older adults care, such as inaccurate health management, lack of personalized and diverse services, and inefficient resource allocation. These advancements have contributed to improving the quality of life for older adults in nursing homes. However, new challenges have emerged, including the ‘digital divide,’ data misuse, and privacy breaches. To fully leverage the benefits of information technology, it is crucial to enhance the digital literacy of disabled older adults, provide robust technical support during implementation, and prioritize data security and privacy protection. These measures will help maximize the positive effects of information technology on the quality of life of disabled older adults.

## 1 Introduction

China entered an aging society in 2000. Although it was not among the first countries to experience population aging, the speed of aging in China ranks among the fastest globally (1). By the end of 2023, China had 297 million individuals aged 60 years and above, accounting for 21.1% of the total population (2). As the country with the largest older adults population in the world, China is characterized by both the vast scale and rapid pace of its aging process. Since 1949, China has experienced three major baby booms: the early 1950s (encouraged childbirth), the mid-1960s (economic recovery), and the late 1980s (a post-reform fertility rebound). As the cohorts born during the second and third baby booms age, the pace of population aging in China is expected to accelerate even further. According to the 2024 revision of the World Population Prospects (United Nations), by 2050, the population of individuals aged 60 years and above in China will exceed 500 million, accounting for approximately 40% of the total population—a near doubling of the current proportion (3).

While China’s older adults population is growing rapidly, its internal structure is undergoing significant changes. With rising living standards and advancements in medical technology, the average life expectancy in China has been steadily increasing, and the older adults population is entering a phase of advanced aging. In the coming years, both the number of individuals aged 80 years and above and their proportion of the total older adults population are projected to grow concurrently (4). However, an increase in the advanced-age population is accompanied by an exponential rise in the risk of disability and semi-disability (5). According to the *Fifth Sample Survey on the Living Conditions of the Elderly in Urban and Rural China*, approximately 35 million older adults in China currently live with disabilities, accounting for 11.6% of the older adult population. This number is expected to rise to 46 million by 2035 and 58 million by 2050 (6). The saying “one disabled person destabilizes the whole family” underscores the significant caregiving demands faced by families of disabled older adults.

China’s fertility rate continues to decline, with 9.56 million births in 2022 and 9.02 million in 2023, marking two consecutive years below the 10 million threshold (2). Factors such as an extended duration of education, urbanization, higher female employment rates, and social attitudes contribute to this trend, which is unlikely to reverse in the short term (7). Low fertility not only constrains the future labor supply but also accelerates population aging, presenting severe challenges to the traditional family-based older adults care model. At present, older adults care in China follows the “9,073” structure, whereby 90% of older adults individuals rely on home-based care, 7% on community-based care, and 3% reside in institutional care facilities. By the end of 2023, 67% of older adults in nursing homes in China were disabled (8). With a shrinking labor force and increasing caregiving demands of advanced-age disabled older adults, the traditional labor-intensive older adults care model is becoming increasingly unsustainable. This makes exploring innovative approaches for the care of older adults an urgent necessity.

In recent years, emerging technologies such as artificial intelligence (AI), big data, cloud computing, and the Internet of Things (IoT) have developed rapidly, establishing themselves as strategic foundational industries in the Chinese economy. With policy support and market-driven demand, the application of information technology in nursing homes in China has expanded significantly. This includes areas such as health management (e.g., wearable devices and health monitoring equipment), older adults assistance (e.g., electric transfer devices and walking robots), older adults care monitoring (e.g., smart monitoring and caregiving devices), digitalized traditional Chinese medicine (TCM) services, home smart services (e.g., smart voice assistants), and age-friendly renovations (9). Studies indicate that 79.69% of nursing homes in China have adopted information technology to support healthcare and daily living (10).

The World Health Organization (WHO) defines quality of life as an individual’s subjective perception and evaluation of their life status within the context of their cultural and value systems based on personal standards, expectations, and concerns (11). As the application of information technology in nursing homes in China continues to expand, its impact on the quality of life of disabled older adults is growing. However, current research on the application of information technology in nursing homes in China primarily focuses on four key areas (10, 12, 13). The first area concerns technology application and product development, including smart medical products, such as portable health monitoring devices and data management platforms, and related services, such as chronic disease management and fall monitoring. Additionally, efforts have been made to integrate third-party platforms with community health service centers. The second area involves health monitoring and management technologies, focusing on real-time monitoring systems that include indoor positioning, vital sign tracking (e.g., heart rate and blood glucose levels), and alert mechanisms for abnormal conditions. The third area examines technology acceptance and user behavior, exploring how older adults adopt information technology products and services, along with the barriers and factors that influence their use. Finally, the fourth area focuses on service model innovation and multi-stakeholder collaboration, emphasizing the need to coordinate resources from government agencies, enterprises, communities, and families to support the effective use of technology in older adults care.

Despite these advancements, research on the impact of information technology on the quality of life of disabled older adults in nursing homes in China remains insufficient. Specifically, three major research gaps persist. First, most existing studies emphasize the perspective of care providers, particularly in the development and implementation of technology products and services, while less attention has been given to the experiences and needs of disabled older adults. Second, there is a lack of comprehensive research examining how China’s unique cultural context—including the government-led collectivist governance model and the modernization of filial piety—affects the adoption and effectiveness of information technology in nursing homes. Third, previous studies have primarily centered on health management technologies, with limited exploration of more heterogeneous aspects, such as how information technology directly impacts the overall quality of life of disabled older adults. Addressing these gaps is essential for providing deeper empirical insights and advancing the understanding of technology-driven improvements in older adults care.

Previous studies have shown that due to physical limitations, increased psychological stress, and insufficient social support, disabled older adults are more sensitive to environmental changes, making their quality of life more vulnerable to the impact (14). As a key population in nursing homes, the quality of life of disabled older adults is directly tied to the overall improvement of caregiving quality.

Given these gaps, this study seeks to address the following research questions:

*RQ1*. How do disabled older adults in Nursing Homes in China experience the use of information technology-based products and services?

*RQ2*. How does information technology impact the quality of life of disabled older adults in Nursing Homes in China?

*RQ3*. What role do China’s unique cultural and social factors play in shaping the impact of information technology on the quality of life of disabled older adults?

Therefore, examining the impact of information technology applications on the quality of life of disabled older adults in nursing homes in China is of critical academic and practical significance. This study not only contributes to the refinement of smart older adults care models, policy development, and technological advancements but also addresses the existing gap in empirical research on this topic. Furthermore, it provides a new analytical perspective and a foundation for future scholarly exploration.

## 2 Methods

### 2.1 Study design

This study employed an interpretative phenomenological design. Such a design aims to explore the reasons behind the existence of certain phenomena, focusing on their ontological nature (15). Given the limited research on the impact of information technology on the quality of life of disabled older adults in nursing homes in China, this study utilized semi-structured interviews to explore and interpret their experiences and the processes involved in using information technology, as well as its effects on their quality of life.

### 2.2 Setting and participants

This study was conducted in Chongqing, China, with participants being disabled older adults receiving care in integrated medical and nursing care institutions. The inclusion criteria were as follows: aged 60 years or older; residing in the institution for at least 6 months; classified as having mild or greater levels of disability according to the *Chinese Specification for the Ability Assessment of the Elderly* (15); and having received care services involving information technology. The exclusion criteria were as follows: severe mental or cognitive impairments, language communication barriers, and physical frailty that prevented participation.

Throughout the design, implementation, and analysis phases of the study, careful attention was given to the research objectives, representativeness of the sample, theoretical framework, interview quality, and appropriateness of data analysis methods. Data saturation was reached after interviews with 12 participants, as no new information emerged. To further confirm saturation, two additional interviews were conducted, and no new themes were identified. Therefore, the final sample size was determined to be 14 participants, all of whom voluntarily agreed to participate in the study.

### 2.3 Data collection

This study was conducted between July and December 2023. The semi-structured interview guide was developed in alignment with the research objectives through discussions among the research team members. Following pilot interviews with three disabled older adults, the semi-structured interview guide was refined and finalized with questions, which are provided in Appendix A1. The semi-structured interviews were conducted by two trained researchers to collect data. Both researchers had received comprehensive training in qualitative research methodologies and had previous experience conducting semi-structured interviews. They were proficient in the fundamental principles and techniques of interviewing and maintained a relatively egalitarian relationship with the participants who were disabled older adults, thereby minimizing potential bias and ensuring data reliability. After obtaining informed consent, the researchers built rapport with the participants through routine communication. Once relevant sociodemographic data and information on the participants’ use of information technology were collected, the formal interviews were conducted and audio-recorded. Throughout the interviews, non-verbal cues such as body language and emotional reactions were systematically recorded in research memos. The audio recordings were transcribed into textual data on the same day as the interview, and these transcriptions, alongside the research memos and the researchers’ reflections, were consolidated into detailed interview notes. Participants were assigned identifiers based on the order of the interviews, and any personally identifiable information (e.g., nicknames) was anonymized. All interviews were conducted in private, quiet, and independent rooms within the nursing home to maintain confidentiality and ensure an environment conducive to open communication. Each interview lasted between 22 and 35 min (*x* = 32 min). Following each interview, the researchers observed and documented the usage of information technology-related products and services in the participants’ rooms.

### 2.4 Data analysis

Van Manen argues that phenomenology is fundamentally interpretative, and therefore, this study applied his approach to data analysis (16). The transcribed data were first numbered in the order of participant enrollment, from D1 to D14. The textual data and interview notes were then imported into NVivo 11.0 software. The analysis proceeded through several steps: developing a holistic understanding of the data, identifying meaningful units, grouping similar units, conducting thematic analysis, establishing and clarifying relationships between themes, and returning to participants to verify the information.

### 2.5 Rigor

Trustworthiness reflects the rigor of qualitative research. This study employed the following strategies to enhance trustworthiness (17): (1) Ensuring researcher credibility: The researchers had both theoretical and practical expertise in qualitative research. They also engaged in continuous self-reflection throughout the study, repeatedly reviewing the interview notes and textual data to ensure that the themes identified were objective and accurate; (2) Using data triangulation: The data collection and analysis process incorporated not only verbal information but also non-verbal data and interview notes, which were informed by the researchers’ reflections; (3) Implementing peer review: Bi-weekly team meetings were held throughout the study, comprising two professors, two associate professors, and seven team members. During these meetings, discussions were held on the development of the semi-structured interview guide, data collection procedures, and the identified themes, which served as supervision and guidance for the design, implementation, and data analysis processes; (4) Using member checking: After the data analysis and theme formulation, the results were returned to two study participants for verification. Both participants confirmed the findings with no objections; (5) Employing in-depth description: Efforts were made to present the relevant data in as much detail as possible throughout the study, including the data collection process, context, and other relevant factors. This approach ensured transparency and enhanced the study’s credibility.

### 2.6 Ethical considerations

This study was approved by the Ethics Committee of the First Affiliated Hospital of Chongqing Medical University (Approval No.: 2023–035). Informed consent was obtained from all participants before the study began. Throughout the data collection and analysis processes, the security and confidentiality of all data were rigorously ensured, with access restricted to the members of the research team. This study adheres to the principles outlined in the Declaration of Helsinki.

## 3 Results

### 3.1 Participant characteristics

A total of 14 disabled older adults participated in the interviews. The participants, identified as D1 to D14, included eight men and six women, with ages ranging from 68 to 91 years (mean age: 82.79 years). Among the participants, seven were classified as having mild disabilities, three with moderate disabilities, and four with severe disabilities. The detailed demographic information is shown in Table 1.

**Table 1:** Demographic characteristics of disabled older adults (*n* = 14).

No.	Gender	Age	Education	Occupation	Marriage	Medical insurance	Payment method	Degree of disability	Length of stay	Numbers of chronic diseases	Offspring	Child visiting frequency	Accomodation
D1	Male	91	Junior college and above	Professional and technician	Widowed	Urban residents basic medical insurance	Pension	Severe	3-5 yr	1–3	Yes	Irregular	Live with other seniors
D2	Male	79	High school/technical school/secondary school	Professional and technician	Married	Self-pay	Pension	Mild	1-3 yr	4–7	Yes	Less than once a month	Live with other seniors
D3	Female	80	Junior college and above	Professional and technician	Widowed	Urban employee basic medical insurance	Pension	Severe	1-3 yr	1–3	Yes	Less than once a month	Live with other seniors
D4	Female	82	High school/technical school/secondary school	Professional and technician	Widowed	Urban employee basic medical insurance	Pension	Moderate	Less than 1 yr	1–3	Yes	Irregular	Live with other seniors
D5	Female	82	High school/technical school/secondary school	Professional and technician	Married	Urban employee basic medical insurance	Pension	Severe	Less than 1 yr	4–7	Yes	Once a month or more	Live with other seniors
D6	Male	88	Junior college and above	Government official/enterprise principal	Married	Urban residents basic medical insurance	Pension	Mild	3-5 yr	4–7	Yes	Less than once a month	Live with other seniors
D7	Female	83	Junior college and above	Government official/enterprise principal	Widowed	Self-pay	Pension	Mild	Less than 1 yr	4–7	Yes	Less than once a month	Live with other seniors
D8	Male	75	Primary school	Professional and technician	Married	Urban residents basic medical insurance	Pension	Mild	More than 5 yr	1–3	Yes	Less than once a month	Live with other seniors
D9	Male	80	Junior college and above	Professional and technician	Widowed	Urban residents basic medical insurance	Pension	Mild	More than 5 yr	1–3	Yes	Less than once a month	Live with other seniors
D10	Male	68	Primary school	Professional and technician	Married	Self-pay	Pension	Moderate	More than 5 yr	4–7	Yes	Less than once a month	Live with other seniors
D11	Male	85	Junior college and above	Professional and technician	Married	Urban residents basic medical insurance	Pension	Severe	3-5 yr	4–7	Yes	Irregular	Live with other seniors
D12	Female	89	High school/technical school/secondary school	Professional and technician	Married	Urban employee basic medical insurance	Pension	Mild	Less than 1 yr	1–3	Yes	Once a month or more	Live with other seniors
D13	Male	91	Junior college and above	Professional and technician	Married	Urban residents basic medical insurance	Pension	Mild	3-5 yr	1–3	Yes	Irregular	Live with other seniors
D14	Female	86	Primary school	Government official/enterprise principal	Married	Urban residents basic medical insurance	Pension	Moderate	More than 5 yr	4–7	Yes	Less than once a month	Live with other seniors

### 3.2 Identified themes

**Figure 1:**
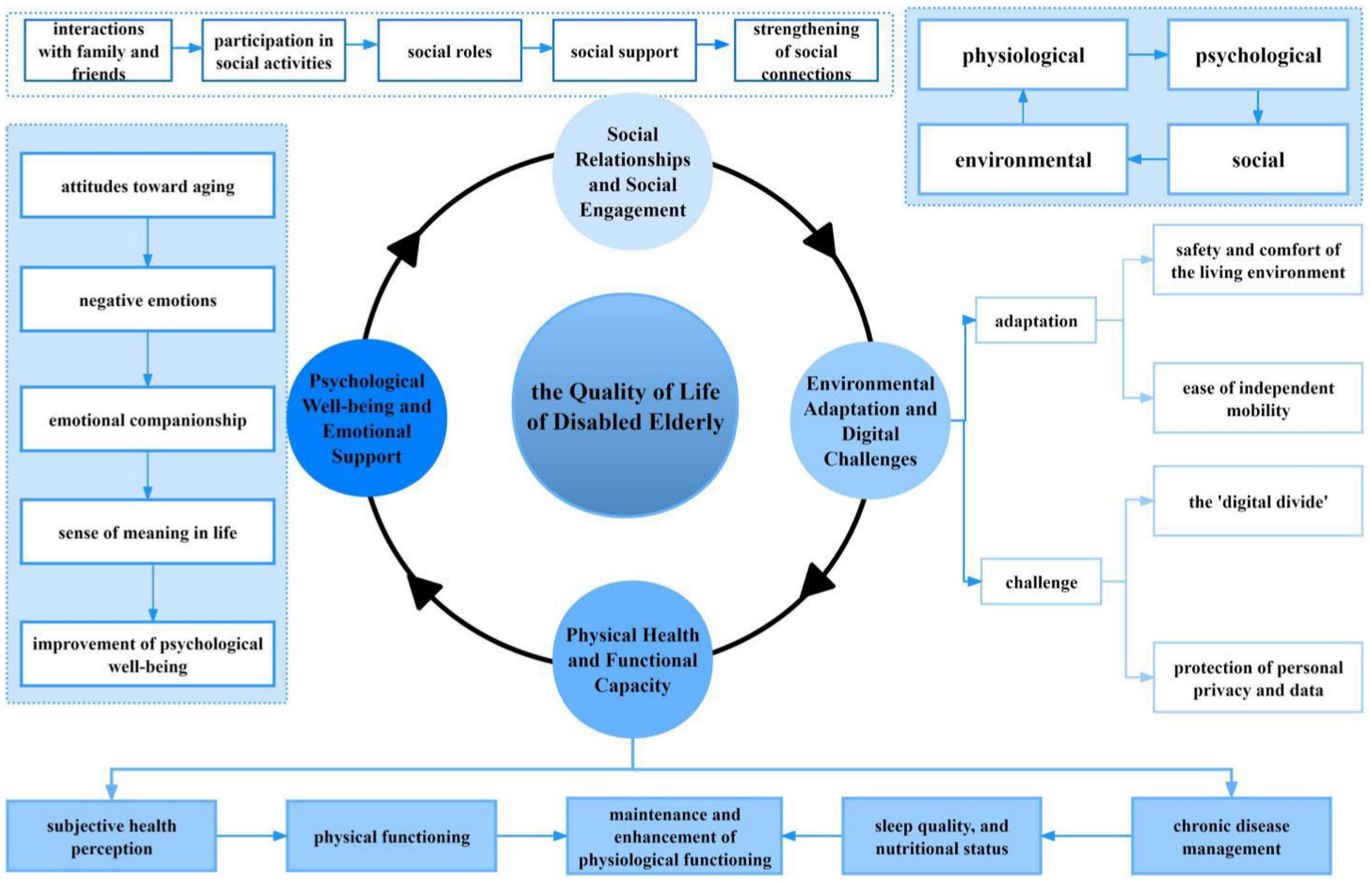
The impact of information technology applications on the quality of life of disabled older adults in nursing homes.

This study analyzed the impact of information technology on the quality of life of disabled older adults in nursing homes in China across four dimensions: physical, psychological, social, and environmental. The identified themes include physical health and functional capacity, psychological wellbeing and emotional support, social relationships and social engagement, and environmental adaptation and digital challenges. The relationships between the themes and sub-themes are shown in Figure 1.

#### 3.2.1 Physical health and functional capacity

##### 3.2.1.1 Sub-theme 1: Subjective health perception

Subjective health perception refers to an individual’s personal assessment of their own health status, which is closely linked to their lifestyle, behavioral choices, and health outcomes (18). Individuals with a positive subjective health perception can interpret their health status optimistically, even when faced with illness or uncertainty, and generally report a higher health-related quality of life (19). Insufficient long-term care support can accelerate the decline in health among disabled older adults, especially in settings where medical resources are limited or caregiving support is insufficient, which significantly exacerbates their negative health perceptions (20).

Health monitoring devices, such as wearable devices and intelligent health monitoring systems, allow for real-time tracking of physiological indicators, providing disabled older adults with a clearer understanding of their health status.

*“Since the nursing home provided me with a smart wristband, I can now monitor my blood pressure and heart rate at any time. The doctors can also access the data in real time and alert me to any health concerns. I feel more in control of my physical condition.” (D6)*

Intelligent caregiving systems optimize resource allocations and provide personalized services, which indirectly improve positive health perceptions.

*“The care system automatically records my health data, so caregivers no longer need to measure it manually every day, significantly improving efficiency. Whenever there is an anomaly in the data, they can detect and address it promptly, which gives me great peace of mind.” (D3)*

##### 3.2.1.2 Sub-theme 2: Physical functioning

As individuals age, the decline in muscle mass, reduced bone density, and the cumulative effects of chronic diseases contribute to the accelerated deterioration of physical functions in disabled older adults (21). Additionally, in Chinese nursing homes, older adults are often restricted from leaving designated areas or engaging in certain activities for “safety” reasons. These restrictions limit opportunities for physical activity, further exacerbating the decline in physical functions (22). Traditional rehabilitation exercises in these institutions are generally low-intensity and non-personalized (e.g., finger exercises), offering insufficient support for the physical activity needs of long-term bedridden disabled older adults.

Health monitoring devices track activity data in real time and issue reminders when prolonged periods of sitting or lying down are detected. Caregivers can intervene promptly to assist or remind older adults to engage in effective physical activities.

*“I used to sit all day without moving, but now the smart wristband reminds me to stand up and move after sitting for too long, encouraging me to do some simple exercises.” (D4)*

Through health management and telemedicine platforms, professional doctors provide regular remote guidance to disabled older adults, adjusting rehabilitation programs to improve the precision and continuity of their exercises.

*“Doctors provide regular guidance for my rehabilitation training through the telemedicine platform and adjust the exercises based on my health data. As a result, my workouts are now more targeted.” (D10)*

##### 3.2.1.3 Sub-theme 3: Chronic disease management

The prevalence of chronic diseases among disabled older adults in Chinese nursing homes exceeds 80% (23). Due to the interplay of multimorbidity, functional impairments, and mental health issues, the chronic disease management needs of these individuals are highly complex and continuous, requiring the integration of multidisciplinary healthcare services. Although China has actively promoted the integration of medical care and elder care at the policy level, most nursing homes lack access to professional medical resources. As a result, older adults with medical needs frequently have to visit external medical institutions, which not only increases the burden of seeking care but may also lead to delays in treatment.

Traditional chronic disease medication management relies on manual record-keeping and experience-based judgments, which increase the risk of improper medication use.

*“I used to forget to take my medicine or accidentally take the morning dose at night. Now, the smart medicine box reminds me and automatically dispenses the medication, so I no longer have to worry about taking the wrong dose.” (D2)*

Video consultations and online medical services provide convenient healthcare solutions for disabled older adults, enabling chronic disease management and health education to be delivered within the institution itself.

*“It used to be very troublesome to go to the hospital when I wasn’t feeling well. Now, I can consult a doctor directly via video in the nursing home. The doctor gives me advice on what to do, saving me a lot of trouble.” (D5)*

##### 3.2.1.4 Sub-theme 4: Sleep quality and nutritional status

In Chinese nursing homes, 67.3% of older adults experience sleep problems (24), 5.1% suffer from malnutrition, and 55.6% are at risk of malnutrition (25). Poor nutritional status has a significant impact on sleep quality, while low sleep quality further compromises the physical and psychological wellbeing of older adults (21). Many nursing homes in China manage residents’ daily routines and dietary arrangements through ‘standardized’ processes, such as fixed routines and uniform meal plans. While this approach improves management efficiency to a certain extent, it often neglects individual differences in residents’ needs.

IoT devices, such as smart monitoring mattresses, enable real-time tracking of sleep patterns, allowing caregivers to identify sleep disorders and implement timely interventions.

*“Since the nursing home installed smart monitoring mattresses, my sleep patterns are recorded daily. Caregivers adjust the room environment based on the data, and as a result, I sleep much better than before.” (D7)*

Intelligent dietary management systems analyze the nutritional needs of disabled older adults based on their health conditions and develop personalized meal plans, addressing the limitations of the “one-size-fits-all” approach of traditional dietary services.

*“Previously, everyone ate the same meals, some of which were unsuitable for me. Now, my diet is adjusted according to my health condition.” (D12)*

#### 3.2.2 Psychological well-being and emotional support

##### 3.2.2.1 Sub-theme 1: Attitudes toward aging

After entering nursing homes, older adults’ attitudes toward institutional care services may improve as they adapt. However, for disabled older adults, physical decline and a high degree of dependence on caregiving often lead to a reduced sense of self-worth, resulting in feelings of “uselessness” and more negative attitudes toward aging (26). Influenced by traditional Chinese filial piety culture, high-quality caregiving is often understood as “replacing” older adults in performing daily activities as much as possible, a practice particularly prevalent among disabled older adults. However, excessive “replacement” can reduce their sense of self-efficacy, further intensifying their negative attitudes toward aging (27).

Intelligent assistive technologies help disabled older adults perform basic daily activities, such as turning over and adjusting their posture, thus reducing their direct reliance on caregivers.

*“I used to rely on caregivers to help me turn over, but now the smart care bed automatically does it for me, so I no longer have to trouble others all the time.” (D6)*

Voice assistants enable disabled older adults to achieve a higher level of autonomy in choosing and controlling their lifestyle by offering hands-free voice control and intuitive human–machine interaction.

*“Now, all I have to do is say ‘Xiaodu, Xiaodu’ (voice assistant), and I can close the curtains, turn on the lights, or play music. It makes me feel like I can still control my life.” (D7)*

##### 3.2.2.2 Sub-theme 2: Negative emotions

The prevalence of negative emotions among disabled older adults in nursing homes in China is notably high, often manifesting as depression, loneliness, and anxiety (28). Although nursing home administrators are increasingly recognizing the adverse effects of negative emotions on residents’ quality of life, the existing management model mainly focuses on physical health while neglecting systematic psychological health interventions. This is largely due to a shortage of professional personnel and cost constraints. As a result, negative emotional issues often remain unaddressed, further exacerbating emotional distress among older adults (29).

Virtual reality (VR) technology, through immersive experiences, provides disabled older adults the opportunity to “return” to familiar environments or explore new settings. This can help to mitigate feelings of loneliness and emotional isolation caused by physical functional decline.

*“I used to just lie there doing nothing, but now, with VR glasses, I can ‘take’ a walk around West Lake. It feels like I can go outside again, and my mood has improved significantly.” (D3)*

Remote psychological counseling offers professional interventions, providing efficient and convenient emotional management support for disabled older adults.

*“I have difficulty moving, but I can communicate with a psychologist through my phone. They teach me how to manage my emotions, and gradually, I’ve become less anxious.” (D11)*

##### 3.2.2.3 Sub-theme 3: Emotional companionship

Compared to self-sufficient older adults, disabled older adults in nursing homes experience significantly reduced social interactions, which increase their psychological vulnerability and heighten their need for emotional companionship (20). According to *China’s Standards for Staffing and Personnel Allocation in Nursing Homes*, nursing homes are required to have a certain number of full-time caregivers based on the number of residents. The recommended caregiver-to-resident ratios are 1:8 to 1:12 for partially disabled older adults and 1:3 to 1:5 for fully disabled older adults (30). However, with only approximately 500,000 caregivers currently employed in China, there is a severe imbalance in caregiver-to-resident ratios. This shortage of caregivers not only increases their workload but also directly contributes to the lack of emotional companionship for older adults.

Social robots, through emotional interactions and companionship behaviors, can engage with disabled older adults by talking, playing games, or providing entertainment activities, thereby enhancing their emotional satisfaction.

*“Even though it’s a robot, it listens to me and makes me laugh. It’s much better than being alone, and I do not feel lonely anymore.” (D1)*

Voice assistants can provide daily companionship and psychological support for disabled older adults through personalized interactions and instant responses.

*“Talking to ‘Xiaodu’ (voice assistant) every day has become a habit for me. It reminds me to take my medicine and tells stories or plays music based on my interests.” [SIC](D9)*

##### 3.2.2.4 Sub-theme 4: Sense of meaning in life

The sense of meaning in life consists of two dimensions: the presence of meaning and the pursuit of meaning (31). The presence of meaning refers to the perception of life’s significance and value, while the pursuit of meaning describes the active process of seeking and constructing one’s purpose in life. Functional disabilities lead to a decline in the ability of older adults to independently manage their daily activities, resulting in feelings of helplessness and dependency. This, in turn, reduces their expectations for the future and impairs their ability to set life goals. Some disabled older adults may even develop negative attitudes toward life, perceiving it as “meaningless” or “hopeless.”

Health monitoring devices collect real-time physical data from older adults and upload it to management platforms. Disabled older adults can access this data and receive timely health advice, thereby fostering a sense of participation and belonging.

*“I can see my health data every day and know what I need to pay attention to. It feels like I’m actively contributing to my own health, rather than just passively receiving care.” (D2)*

The application of information technology can enhance the sense of being cared for among disabled older adults by facilitating efficient communication and personalized services.

*“Previously, I had to wait for caregivers to check on me when I needed something. Now, I can simply speak to the voice assistant to reach them.” (D10)*

#### 3.2.3 Social relationships and social engagement

##### 3.2.3.1 Sub-theme 1: Interactions with family and friends

Regular interactions with family members and friends are essential for older adults to maintain their social connections with the outside world. Study shows that approximately 60% of disabled older adults receive only one to two visits per month, while over 30% are visited just once per quarter (32). These infrequent visits are primarily due to factors such as the closed management practices of nursing homes, work-related pressures on family members, geographical distance, and intergenerational allocation strategies that prioritize children’s needs while addressing older adults’ care primarily during crises. The low frequency of visits further reduces opportunities for disabled older adults to receive direct support from their families.

Technologies such as video calls and social media platforms can overcome the constraints of time and space, increasing the frequency of interactions between disabled older adults and their family members and friends.

*“My children used to be too busy with work to visit me often, but now, with video calls, I can see them every week. It feels like they are right here with me.” (D12)*

Smart devices, such as tablets and smartphones, allow family members to monitor the condition of disabled older adults in real time, enabling non-intrusive remote interaction and support.

*“My blood pressure is visible to my children on their phones, and they often make video calls to check on my health. It feels like they are always looking out for me.” (D6)*

##### 3.2.3.2 Sub-theme 2: Participation in social activities

Social activities are vital for maintaining social relationships among disabled older adults. In Chinese nursing homes, activities such as arts and crafts, fitness and entertainment programs, themed parties, and community events are commonly organized. However, these activities are primarily designed for older adults with greater self-care capabilities. While disabled older adults face social barriers due to their physical limitations, they demonstrate a strong desire to engage in social interactions and aspire to participate in these activities through appropriate means (20). Unfortunately, the design of activities in nursing homes in China often lacks inclusivity, overemphasizing participation by those with full physical functionality while overlooking opportunities for disabled older adults to engage through assistive devices or alternative methods.

By using technologies such as smart tablets and video conferencing software, nursing homes in China can organize online birthday celebrations, remote family gatherings, and virtual community cultural events, effectively addressing mobility limitations and alleviating feelings of social isolation.

*“The nursing home organizes online activities for us using tablets. I’ve met many new friends, and life has become much livelier than before.” (D7)*

*“Although I have mobility issues, I can participate in online calligraphy classes and interact with other older adult individuals. It feels like I’m part of a community again.” (D13)*

##### 3.2.3.3 Sub-theme 3: Social roles

Social roles refer to the specific identities and behavioral patterns that individuals adopt within social relationships and group activities. Before entering nursing homes, disabled older adults often held roles within their families, such as decision-makers, emotional supporters, and inter-generational connectors. In society, they might have been economic contributors or community participants. However, after moving into nursing homes, physical limitations, reduced social interactions, and the institutionalized management of the nursing home environment gradually weaken or even eliminate these roles. Instead, they are replaced by the passive identity of a “care recipient,” leading to a decline in their sense of identity and self-esteem (33).

Digital technologies, such as virtual interaction platforms, video calls, and online communities, can effectively strengthen the connections between disabled older adults, their families, and their communities, helping them reintegrate into social networks.

*“Now I can participate in online community activities, discuss topics with other older adult people, and even give advice to younger generations.” (D14)*

By using assistive technologies (such as voice-controlled devices or decision support systems), disabled older adults can re-engage in family decision-making, thereby enhancing their sense of self-identity.

*“During video consultations, doctors discuss rehabilitation plans with my family, and I can also share my opinions. This makes me feel like I’m still an important member of the family.” (D10)*

##### 3.2.3.4 Sub-theme 4: Social support

Social support, defined as the emotional, informational, and material resources obtained through social networks, plays a crucial role in individuals’ mental health and quality of life. However, traditional nursing homes primarily depend on internal resources and have limited connections with external social networks, restricting disabled older adults from receiving comprehensive social support (34). Information technology can facilitate the integration of medical, community, and family resources, offering multidimensional support to disabled older adults and improving both the breadth and depth of their access to social support.

Virtual interaction platforms can enable real-time communication and online community engagement, helping disabled older adults access emotional resources.

*“I used to feel disconnected from the outside world, but now, through online platforms, I can not only stay in touch with my family anytime but also make new friends.”(D1).*

Telemedicine, through the integration of medical resources, can provide disabled older adults with continuous health guidance and informational support.

*“Through remote consultations, doctors helped me adjust my medication and taught me how to exercise. Even though I have mobility issues, my health concerns can now be addressed in a timely manner.” (D11)*

#### 3.2.4 Environmental adaptation and digital challenges

##### 3.2.4.1 Sub-theme 1: safety and comfort of the living environment

Safety issues in the living environment of nursing homes include physical hazards, such as slippery floors, inadequate lighting, and inappropriate bed heights, as well as delays in response caused by a lack of caregiving staff (35). Disabled older adults, owing to factors such as reduced mobility and cognitive decline (e.g., weakened attention and judgment), are more sensitive to such environmental risks. They experience a higher frequency of adverse events, such as falls, compared to self-sufficient older adults, with longer recovery times and more severe consequences (36).

Technologies such as sensor monitoring systems and remote alarm devices can provide real-time tracking of the activities of disabled older adults, allowing for the timely detection of safety hazards, such as falls.

*“One time, I slipped and fell in my room. The sensor immediately triggered an alarm, and the caregiver quickly came to help me up. Fortunately, I wasn’t injured.” (D1)*

Smart lighting and temperature control devices can automatically adjust the environment to meet the needs of disabled older adults, thereby enhancing their comfort and safety.

*“The lights in my room automatically adjust to my needs. At night, I do not have to fumble in the dark to find the switch. It feels very convenient and safe.” (D7)*

##### 3.2.4.2 Sub-theme 2: Ease of independent mobility

Disabled older adults often require varying levels of assistance from caregivers to complete daily activities due to a decline in their physical abilities. However, such excessive dependence not only increases the workload of caregivers but also restricts the use of these individuals’ “remaining abilities” (37). In China, nursing homes frequently use physical restraint measures for disabled older adults to prevent falls, address caregiver shortages, and compensate for the lack of alternative solutions. While these measures may reduce accidents in the short term, their long-term use accelerates physical functional decline and leads to psychological issues such as depression and anxiety (38).

Intelligent assistive technologies can effectively support disabled older adults perform daily activities, enhancing their independence.

*“The smart care bed allows me to adjust my sitting position on my own, making activities like eating or reading much more convenient. I no longer have to call for help every time.” (D3)*

Smart wheelchairs, equipped with automatic obstacle avoidance and path planning, can help disabled older adults efficiently complete indoor and outdoor movements.

*“I used to worry about bumping into things with a regular wheelchair, but now this one automatically avoids obstacles. It’s stable and safe.” (D10)*

##### 3.2.4.3 Sub-theme 3: “digital divide”

Disabled older adults in nursing homes face a significant “digital divide” in the adoption of information technology. This issue is primarily characterized by their insufficient digital skills, the high complexity of technology, and a lack of technical support, all of which are closely associated with their age, level of education, and physical functional decline (39). The “digital divide” not only hinders disabled older adults from accessing smart care services but may also exacerbate health inequalities within institutions. It can result in a skewed allocation of resources toward individuals who are more proficient with technology, further marginalizing disabled older adults.

Some disabled older adults fail to recognize the potential benefits of information technology, perceiving traditional methods as more familiar and reliable.

*“I feel like these things are useless. I do not understand them anyway, and I’m more accustomed to the old ways.” (D8)*

The complexity of information technology, coupled with the lack of proper education and support, also makes it challenging for disabled older adults to actively adopt new technologies.

*“Sometimes I want to use it, but I do not know how. If I press the wrong button, there’s no one to help me, so I end up giving up.” (D4)*

##### 3.2.4.4 Sub-theme 4: Protection of personal privacy and data

Care services related to information technology require the collection and processing of substantial amounts of personal and health data from disabled older adults to improve service efficiency and accuracy. However, in practice, data management systems in nursing homes are often inadequate, staff training in data security is insufficient, and legal and regulatory frameworks are incomplete. These deficiencies increase the risk of sensitive information being leaked or misused during storage and transmission, as well as the likelihood of internal data breaches (9). Such challenges not only undermine the trust of disabled older adults in information technology but may also reduce their willingness to adopt these technologies.

Insufficient privacy protection is a key reason for mistrust of information technology among disabled older adults.

*“These machines are always recording my information. I’m not sure if this data will be used elsewhere, and it makes me feel uneasy.” (D11)*

Concerns about the storage and use of health data are also widespread, particularly fears that data breaches could result in discrimination or unfair treatment.

*“Although these devices are convenient, I’m always worried that my medical condition might be exposed. If others find out, will they judge me? I feel it’s safer not to use them.” (D14)*

## 4 Discussion

In recent years, the smart care sector for older adults in China has accelerated its transition toward digitalization and intelligence by leveraging advanced information technologies, including AI, IoT, and big data. Nursing homes, as key implementation sites for these technologies, have been increasingly integrating them into daily caregiving practices. Due to their inherent “vulnerability,” disabled older adults are particularly sensitive to the adaptation process of IT-based care services, making their quality of life more susceptible to change. In this context, exploring the impact of information technology on the quality of life of disabled older adults in nursing homes in China has become a critical issue in addressing the challenges of an aging society.

This study has analyzed the impact of information technology from four perspectives: physical, psychological, social, and environmental. The findings reveal that information technology has significant potential to address the limitations of traditional caregiving models and enhance the quality of life for disabled older adults. However, as the application of these technologies remains in its early stages, several challenges, such as limited adaptability among older adults, insufficient training resources, underdeveloped technical support systems, and privacy concerns, may introduce new challenges to improving their quality of life.

The physical functioning of older adults is closely linked to their ability to adapt to institutional environments, as well as to their overall quality of life. Good physical functioning not only improves self-care abilities but also enhances psychological resilience and social adaptability (40). Research has demonstrated that improvements in physical function are strongly associated with older adults’ subjective health perception. In the context of chronic disease management, maintaining good physical function has been shown to significantly reduce medical expenses and hospitalization rates (41). Physical decline, influenced by aging, the accumulation of chronic diseases, and the loss of mobility, reduces activity levels and accelerates functional deterioration (41). Studies suggest that among disabled older adults, those who engage in regular physical exercise experience a significantly slower rate of functional decline compared to those who do not, emphasizing the crucial role of physical activity in maintaining functional ability (42). In China, nursing homes primarily focus on basic living care, with insufficient medical resources and challenges in integrating medical and care services for older adults. These include uneven distribution of medical resources, inadequate specialization, and poor institutional coordination, which hinder disabled older adults from accessing timely health management services, increasing the risks of chronic disease progression and further functional decline (43).

Health monitoring devices and intelligent caregiving systems improve disabled older adults’ awareness of their health and enhance their subjective health perception. This aligns with the Health Belief Model, which posits that positive health perceptions promote healthier behavioral choices and improve quality of life (44). Studies show that older adults who use health monitoring devices experience significant improvements in self-management of their health, which in turn enhances their quality of life, particularly in managing chronic disease (45). Despite functional impairments, disabled older adults can maintain or even improve their physical functioning by using residual capabilities through exercise (46). Health monitoring devices, combined with telemedicine platforms, provide precise activity guidance and rehabilitation support, reducing functional decline caused by insufficient activity. Additionally, technologies such as smart medicine boxes and video consultations optimize chronic disease management processes, lowering the burden of seeking medical care and minimizing medication risks. This study further confirms the previous finding that information technology interventions can optimize rehabilitation and chronic disease management for older adults (47) while also emphasizing the importance of personalized interventions.

Moreover, the institutionalized management of nursing homes in China often neglects the personalized sleep and nutritional needs of older adults, resulting in unresolved issues in these areas. By utilizing smart monitoring mattresses and dietary management systems, nursing homes can provide personalized solutions for disabled older adults, effectively avoiding the limitations of the traditional “one-size-fits-all” approach. Research shows that personalized sleep and nutrition management can significantly improve the quality of life for older adults by regulating core aging mechanisms while also considering individual physiological characteristics and lifestyle differences, thereby promoting the synergistic optimization of multiple bodily functions (48). The findings of this study on improvements in sleep and nutritional status align with existing research, highlighting the importance of personalized care in enhancing quality of life (49).

A positive psychological state can enhance the motivation of disabled older adults to participate in physical activities, thereby improving their overall health (29). Research indicates that maintaining a positive attitude toward aging can effectively reduce the incidence of depression and anxiety by enhancing self-efficacy, encouraging social engagement, and mitigating the effects of negative aging stereotypes (50). However, the Chinese culture of filial piety emphasizes comprehensive care, leading caregivers to often “take over” all activities for disabled older adults, which reflects filial piety but ignores the importance of maintaining physical functions and accelerates functional deterioration (51). Although psychological support has been incorporated into China’s care system for older adults, the lack of professional psychological counselors and inadequate mechanisms for monitoring and intervening in psychological problems have resulted in the psychological needs of disabled older adults remaining unmet for a long time (52). Insufficient psychological support not only affects quality of life but may also accelerate the deterioration of physical function, creating a vicious cycle.

Intelligent assistive devices and voice assistants can be used to help disabled older adults achieve a certain degree of autonomy and improve their attitude toward aging. Studies have shown that information technology, by improving the quality of daily life, can prompt older adults to view aging more positively (53). Research suggests that the use of smart devices can significantly improve older adults’ attitudes toward aging and enhance their mental health by facilitating social interaction, reducing feelings of loneliness, providing easy access to health information, and addressing personalized needs (54).

In addition, negative emotional problems are common in nursing homes, where emotions such as depression and anxiety are not effectively intervened in for long periods due to insufficient professional psychological support, which directly affects the older adults’ ability to perform their daily activities, and depressive symptoms can also exacerbate dependence on care (20). VR technology and e-psychological counseling provide innovative means of emotional management for old adults with disabilities, effectively alleviating feelings of loneliness and anxiety. Research indicates that VR technology enhances older adults’ participation in group interactions by offering immersive social activities such as virtual dance clubs and travel experiences while also encouraging active user engagement. Through these experiences, older adults transition from passive technology users to active creators, for instance, by designing virtual gardens and inviting others to visit. Such interactive engagement has been shown to effectively alleviate feelings of loneliness and improve emotional wellbeing (55). This highlights the potential of modern technological interventions in addressing the lack of professional psychological support for older adults.

Due to the shortage of caregivers in China, the social interactions of disabled older adults are reduced, and their emotional needs are difficult to fulfill. Social robots and voice assistants can partially make up for this lack of emotional support through interaction and companionship, enhancing the sense of emotional fulfillment. The sense of meaning in life not only includes the perception of the value of life but also involves the ability to actively construct life goals. A state of incapacitation weakens the sense of meaning in the lives of older adults, who in the traditional care model in China are passive recipients of services and lack a sense of control over their lives (22). Information technology can enhance the sense of participation and belonging of disabled older adults through health monitoring devices and efficient communication platforms, helping them to reestablish meaning in their lives.

Opportunities for social activities for disabled older adults in Chinese nursing homes are generally insufficient, mainly due to these institutions’ excessive focus on safety risks, lack of relevance in the design of activities, and limited communication among disabled older adults (56). Research indicates that strong social support and active social engagement can significantly reduce the risk of negative emotions, such as depression in older adults by providing emotional support, promoting cognitive stimulation, and alleviating feelings of loneliness (57). Moreover, by enhancing psychological wellbeing and cognitive function, social engagement can indirectly improve life satisfaction, which aligns with the observations of this study.

Additionally, family members often reduce the frequency of visits due to work pressures and geographical distance, further weakening the older adults’ connection with the outside world. Although some institutions try to organize activities, the participation rate of disabled older adults is still low due to their limited mobility, cognitive impairment, and the single form of activity, which further exacerbates social isolation. Research suggests that social engagement significantly mitigates the negative impact of perceived health status on depressive symptoms in disabled older adults. In groups with high levels of social engagement, the effect size of perceived health on depression was significantly lower than in groups with low participation. This finding indicates that enhancing the design of social activities can strengthen the moderating role of social engagement, thereby contributing to improved mental health outcomes (58).

Through technological means such as smart tablets and videoconferencing software, disabled older adults can overcome mobility barriers and participate in online social activities, thereby reducing social isolation. This is consistent with the findings of recent global studies on the role of digital technology in alleviating social isolation among older adults (59) and further reinforces this study’s argument regarding the effectiveness of technological intervention.

The application of information technology has also transformed the nature of social engagement. Virtual interest groups, online education courses, and remote volunteering activities can provide personalized opportunities for disabled older adults and help them establish new social connections. Research shows that these emerging forms of participation not only expand older adults’ social networks but also strengthen their sense of social identity (58).

Disabled older adults are at an increased risk of falls or accidents due to the interaction between their physical limitations and potential hazards in nursing home environments, such as uneven floors, narrow corridors, and a lack of anti-slip facilities. Older adults with cognitive impairments, owing to weakened spatial perception and judgment, have even higher demands for environmental adaptability. Research suggests that well-designed environmental safety measures can effectively reduce the incidence of falls and other accidents among older adults by improving environmental adaptability, such as installing non-slip floors, optimizing lighting, and adjusting furniture, thereby significantly enhancing their quality of life (36).

The caregiving model in nursing homes in China is predominantly passive, with an excessive emphasis on service provision while neglecting the independence and autonomy of older adults. While this model may meet short-term needs, it can have several negative long-term consequences, including physical functional decline, reduced self-efficacy, and increased dependence on caregiving (27). This study found that the application of information technology, such as intelligent monitoring devices and emergency call systems, can enhance the adaptability of disabled older adults to their environment, reduce the risk of accidents, and improve their perception of safety and trust. Intelligent monitoring devices and remote alarm systems can monitor the activity status of older adults in real time, effectively preventing safety incidents such as falls, which is particularly critical for disabled older adults who have declining mobility.

A comparative study of older adult communities with and without smart monitoring fall detection systems found that those equipped with such devices experienced a significant decrease in weekly fall incidents (60). Smart lighting and temperature control systems, which dynamically adjust environmental conditions based on individual needs, can significantly improve their living comfort. Intelligent assistive technologies, including smart care beds and smart wheelchairs, can provide disabled older adults with greater autonomy in their activities, thereby reducing their dependence on caregivers.

However, the use of information technology also presents several challenges, such as the “digital divide” and privacy protection. Disabled older adults generally have insufficient operational ability, low acceptance of technology, and anxiety regarding technology application due to aging, low levels of education, and cognitive impairments (9). Complex interfaces and cumbersome operations limit the opportunities for older adults to enjoy the benefits of technology, exacerbating feelings of social isolation and dependence on caregiving services (39).

Although the digital divide, data privacy, and security concerns are prevalent across various information technology applications, their impact is particularly pronounced among the older adult population (61). Research indicates that older adults generally have lower acceptance of new technologies compared to younger individuals. This disparity is influenced by both personal characteristics and levels of trust in technology. For instance, individuals with higher education levels tend to be more open to technological adoption. Additionally, trust in technology is affected by concerns over data security, privacy breaches, and system reliability (62). In many cases, technology-related fear and distrust among older adults stem primarily from concerns about personal privacy and data security. This is especially evident in the use of digital health platforms, where apprehensions about third-party data sharing, identity exposure, or security risks contribute to a more cautious attitude toward technology-related services (63).

In addition, non-standardized data management and insufficient security measures pose risks of privacy breaches and data misuse, further undermining older adults’ trust in and willingness to use technology. In some cases, this can even result in the avoidance of technology-assisted services, thereby restricting the potential of technology to improve their quality of life. According to the Intelligent Systems for Assessment of Aging Changes (ISAAC) survey in the United States, more than 72% of older adult participants accepted home and computer-based monitoring and were willing to share their data with doctors. However, 60% of participants expressed concerns about privacy and security, and these concerns increased after 1 year of participation. Notably, among cognitively normal participants, acceptance of video surveillance declined significantly, dropping from 20 to 7% (64). Addressing the challenges of the “digital divide,” data privacy, and security concerns is not only essential for enhancing older adults’ experience with technology but also plays a significant role in promoting their social engagement and improving their overall quality of life.

In summary, the application of information technology in nursing homes in China has significantly transformed traditional care service models for older adults, greatly improving the quality of life for disabled individuals. However, challenges such as the lack of digital skills and barriers to adopting new technologies remain prevalent among this population. The “digital divide” has hindered the widespread adoption of technology, highlighting the urgent need to enhance digital literacy among older adults through targeted education and training programs while also simplifying technological interfaces and operations. Moreover, concerns about privacy protection and data security have become increasingly prominent. Non-standardized data management erodes older adults’ trust in technology and impedes the broader adoption of technological solutions. Therefore, it is essential to optimize technology design, improve the digital skills of older adults, and establish strong privacy protection mechanisms to create a user-centered information technology system tailored to their specific needs. At the same time, comprehensive legal frameworks and enhanced technological supervision are necessary to ensure data security and maximize the effectiveness of information technology in care services for older adults.

## 5 Strengths and Limitations

This study adopted an interpretative phenomenological approach to explore the subjective experiences and adaptation processes of disabled older adults in nursing homes in China regarding the application of information technology. The aim was to provide a comprehensive understanding of its impact on their quality of life. However, there are several limitations to this study. First, the sample was limited to nursing homes in Chongqing, China, and this regional focus may restrict the generalizability of the findings of the study to other regions or countries. Second, the data sources were relatively singular, relying primarily on the subjective accounts of disabled older adults, without fully incorporating the perspectives of caregivers or nursing home administrators. This limitation may result in a potentially incomplete understanding of the overall impact of the application of information technology in nursing homes in China. Considering the critical role of caregivers in the care process, future research should incorporate interviews with nursing staff and caregivers to develop a more comprehensive understanding of the application of information technology in care services for older adults and its impact on disabled individuals. Additionally, the study’s scope was limited to existing technologies already implemented in nursing homes, leaving the potential contributions of emerging technologies in care services for older adults unexplored. Future research should expand the sample size and integrate multiple perspectives, particularly that of caregivers, to provide a more comprehensive analysis of the impact of information technology on the quality of life of disabled older adults.

## 6 Conclusion

This study, employing an interpretative phenomenological approach, thoroughly examined the impact of the application of information technology on the quality of life of disabled older adults in nursing homes in China. Through an analysis across four dimensions—physical, psychological, social, and environmental—it was found that the application of information technology has partially addressed challenges in traditional care services for older adults, such as imprecise health management, lack of personalization and diversification of services, and inefficient resource allocation. As a result, it has substantially enhanced the quality of life of disabled older adults, including improvements in physical health, psychological wellbeing, social relationships, and environmental adaptability. However, the study also uncovered several challenges, such as the “digital divide,” data misuse, and privacy breaches, which highlights the complexity and multifaceted nature of applying information technology in care services for older adults. Therefore, future efforts should focus on improving the digital literacy of disabled older adults, strengthening technical support and privacy protection, and optimizing age-friendly designs of information technology. It is essential to build a technology application system centered around the needs of older adults to fully maximize the positive effects of information technology on their quality of life. This approach would provide both theoretical foundations and practical directions for the sustainable development of smart care for older adults.

Furthermore, the findings of this study serve as a valuable complement to the existing literature, particularly in understanding the relationship between information technology and the quality of life of disabled older adults. By integrating the results of this study with prior research, a more comprehensive understanding of the effectiveness of information technology in care services for older adults can be achieved, offering new perspectives for future studies. Existing research suggests that the personalized application of information technology can significantly enhance the life satisfaction of older adults (65), and the findings of this study further validate this view, emphasizing the multidimensional impact of technological applications. Therefore, future research can build upon these findings to explore more effective strategies for integrating information technology into care services for older adults to address the growing needs of the aging population.

## Data Availability

The original contributions presented in the study are included in the article/supplementary material, further inquiries can be directed to the corresponding author.

## Appendix 1

The semi-structured interview guide.

**Table:**

What types of information technology-related care services have you received in your daily life at the nursing home? What changes have these information technology-related care services brought to your daily life? In your opinion, what are the key factors for achieving a high quality of life in a nursing home? What specific impacts have these information technology-related care services had on your quality of life?
